# Chapter II: General Conditions of Symptoms of the Head

**Published:** 1889

**Authors:** Edmund J. Lee


### GENERAL CONDITIONS OF SYMPTOMS OF THE HEAD.

**Day, all**: agar. am-c. bry. calc. cann-s. caus. chel. chin-s. cina. cist. cob. coca. eup-per. ferr. fluor-ac. ham. jac. k-ca. lyc. merc-i-r. *nat-m.* nicc. pau-p. phos. rumx. sep. stann. staph.
— — but is worse at mid-day: *nat-m.*
— — **forehead**: caus. chel. con. cund. k-ca. lach. lil-t. lyc. mag-c. nat-m. nuph. petr. phos. ptel. ran-b. sep. sil. sol-t-æ. tarent. znc.
— — **occiput**: carb-v. ign. mag-c. petr. ph-ac.
— — **sides**: cact. ferr. hydr. mag-m.
— — **temple**: ars. calc. h ll. hepar. hydr. hydrph. jatr. mezer. nitr. stann.
— — **vertex**: sep. sulph. tabac.
— **alternate** days: alum. ambr. chin. cimic. nat-m. nx-v. phos.
— — see also under Periodical.
— — at certain hours: nat-c.
— — on first waking: eup-per.
— — every three or four days: aur.
— — every seven days: nx-m. phyt. sang. sil. sulph.

— — every fourteen days: nicc. phyt. sulph.
— — — — — lasting two or three days: ferr-acet.
— **and night**: borax. caus. creos. led. *rhus*. sul-ac. viol-tr.
— **every day**, daily: ars. bell. calc. coloc. con. eup-per. hepar. lach. lyc. mag-c. mag-m. mang. mur-ac. *nat-m*. *nx-v*. petr. phos. sabad. seneg. sep. *sil*. stann. sulph. znc.
— — — at same hour: ars. cimic. gels. k-bi.
— — from 9 to 1 P. M.: mur-ac.
— — from noon to 10 P. M.: form.
— — earlier each day: form.
— — continues two or three days: croc.

**Morning**: acon. *agar*. all-s. alum. ambr. am-c. am-m. anac. ang. ant-t. arg. arg-n. arn. ars. asaf. asar. aur. bar. bell. benz-ac. berb. borax. bov. *bry*. cadm. *calc*. calc-p. camph. cann-s. canth. carb-an. carb-v. caus. cham. chin. chin-s. cic. cina. clem. cob. coca. coff. coloc. con. creos. croc. croton. cund. cupr. cyc. dios. dulc. eup-pur. euphr. ferr. glon. *graph*. grat. guai. hell. *hepar*. hipp. *ign*. indm. iod. ipec. iris. jatr. k-bi. k-ca. k-iod. kalm. lac-can. lach. lachn. lact. led. lil-t. lith. mag-c. mag-m. manc. mang. merc. merc-i-fl. merc-s. mezer. murx. mur-ac. nat-c. *nat-m*. nicc. nit-ac. nitr. nx-j. nx-m. *nx-v*. ol-an. pæon. pallad. pau-p. petr. *phos*. *ph-ac*. phyt. podo. psor. puls. ran-b. rheum. rhod. rhus. rumx. ruta. samb. sang. sars. scut. seneg. *sep*. sil. spig. *squil*. stann. *staph*. stram. stront. *sulph*. sul-ac. tabac. *thu*. verat. znc.
— — better in: bov. caus. creos. nat-m. verat.
— — at 3 A. M.: bov. nat-m. thu.
— — 4 A. M.: chel. raph. stram.
— — 5 A. M.: dios. k-iod.
— — 10 A. M.: nat-m.
— — until 10 A. M.: lachn.
— — 10 A. M. to 6 P. M.: apis.
— — at same hour: k-bi.
— — begins in morning, increases till noon, or a little later, then gradually decreases: sulph.
— — — — — — — — and ceases toward evening: *bry*. k-bi. *nat-m*. sang. spig.
— — increases and decreases with the sun: glon. kalm. nat-m. spig.
— — comes and goes with the sun: k-bi. nat-m.
— **forehead**: agar. alumn. am-m. aster. bell. borax. bov. brom. bry. calc-s. canth. chel. chin-s. cimic. coca. creos. croton. cyc. dios. equ. euphr. ferr. form. hydr. hydrph. junc. k-bi. k-ca. lach. lact. lil-t. lyc. mag-c. mag-s. merl. mezer. murx. naja. nat-c. nicc. nit-ac. nx-m. nx-v. ol-an. oxal-ac. pæon. phos. psor. raph. rhus-r. scut. seneg. sep. sil. stram. *sulph*. tarent. thu. ustil. znc.
— — better in: mag-s. oxal-ac. petr.
— **brain**: k-bi. lach. spig.
— **occiput**: agar. all-s. arum-t. bov. cedr. chin-s. cob. colch. cop. dios. gels. junc. lobel. lyc. mac. mag-c. mag-s. nit-ac. petr. puls. raph. rhod. rhus. rhus-r. sabin. sil. spig. sulph.
— — in room, amel.: bov.
— **sides**: aloe. alum. bell. bov. chin-s. chr-ac. dios. euphr. fluor-ac. gels. graph. ham. hipp. hydr. jug-c. mag-c. mang. sars. *spig*. tabac.
— **temple**: all-c. am-c. apis. bar. camph. clem. cob. coloc. cop. cund. dios. dirc. equ. graph. ham. hydrph. ign. jac. lith. lil-t. nat-ar. nat-p. nitr. phos. rhus. rhus-r. rumx. sang. sulph. tarent. thu.
— — amel. in: mag-s.
— **vertex**: agar. ambr. aster. bov. conv-d. graph. hydr. hyper. iris. lac-ac. merc-s. nat-c. ran-b. staph. sulph. thu.
— — amel. in: laur.

**Morning on waking**: agar. ailan. arg-n. ars. benz-ac. bov. bufo. calc. cann-i. carb-an. caus. cham. chel. chin. cic. cob. coff. colch. con. creos. croc. crotal. croton. cupr-ars. dig. elaps. equ. erig. eup-per. fago. form. graph. hell. hepar. hipp. ign. indm. jug-c. k-ca. k-bi. kalm. lam. lil-t. lobel. lyc. murx. mur-ac. merc-i-fl. myric. *nat-m*. nit-ac. nitr. nx-v. ol-an. op. petiv. phos. ph-ac. phys. pip-m. plan. plect. puls. rhus-r. rumx. sep. squil. stann. staph. sul-ac. tarent.
— — — **aggravated**: benz-ac. crotal. eup-per. thu.
— — — preceded by disagreeable dreams: murx.
— — — on first opening the eyes: *bry*.

— — **forehead**: acon. alumn. alum. anac. ant-t. arg. arg-m. arg-n. arn. bell. berb. bry. calc. carb-an. chin-s. cina. cinnb. coff. colch. coloc. creos. dig. erig. euphr. fago. ferr. fluor-ac. gels. glon. graph. hepar. hydr. ign. indm. k-bi. kalm. lac-ac. lact. lyc. mag-c. mag-m. myric. naja. nat-ars. ol-an. oxal-ac. petr. phos. ph-ac. plect. raph. rhus. rumx. ruta. sang. sol-n. staph. *sulph.* tellur. therid. thu.
— — **occiput**: arn. bry. con. fluor-ac. grat. k-bi. mill. oxal-ac. petr. rhus. sulph. uran.
— — **side**: arum-t. aur. cina. merc-i-r. mur-ac. phos. puls. tabac.
— — **temple**: ailan. asim. atro. calad. calc. camph. cast-eq. coff. indm. lacb. lith. naja. nat-ars. nit-ac. tabac. znc.
— — **vertex**: alum. bar. bry. bufo. calc. carb-an. caus. cedr. croc. hyper. k-bi. puls. *sulph.* tabac. verat.
**Morning in bed**: *agar.* alum. am-c. anac. ant-t. aur. bar. bell. berb. bov. *bry.* calc. calc-p. carb-an. *cham.* chin. chin-s. cic. coff. con. creos. dig. dulc. ferr. graph. hell. hepar. ign. ipec. k-ca. k-iod. lach. lact. lam. laur. lyc. mag-c. mag-m. mag-s. mang. merc. mezer. murx. *nat-m. nit-ac. nx-v.* petr. phos. ptel. ran-b. rheum. rhod. rhus. ruta. squil. staph. sulph. sul-ac. thu. verat. znc.
— — — with nausea: calc. cob. graph. nat-m. nx-v. sep. sil. sulph.
— — **forehead**: anac. dulc. graph. inu. mezer. nx-v. ran-b.
— — **occiput**: agar. eupi. jug-c.
— — **temple**: anac.
— — **side**: graph. nicc. nx-v. scut. spig.
— — **vertex**: carb-v. hell.
**Morning on rising**: am-c. am-m. apis. arg-n. aur-m. bar. bry. camph. chin-s. cob. colch. croton. cyc. dig. dulc. fago. glon. ham. hepar. hydr. indm. ipec. jug-c. kalm. lach. lyc. mag-c. mag-m. merc. mur-ac. nicc. nx-v. petr. phos. psor. ptel. puls. rhus. rumx. sep. squil. staph. stront. sulph. tarent. tong.
— — — amel.: alum. cham. ign. k-iod. merc-i-r. murx. nat-m. nit-ac. nx-v. ph-ac. rhod.
— — **brain**: indg. ped. ruta.
— — **forehead**: am-m. asar. bar-ac. bry. carb-an. cob. con. dulc. ferr. graph. ham. iber. k-bi. kalm. lil-t. lyc. mag-c. nat-c. nat-m. nitr. psor. raph. sep. sil.
— — — amel.: nx-v. phos. ran-b.
— — **occiput**: cimic. cinnb. gels. k-bi. mag-m. merc-i-fl. *nx-v.* ped. tong.
— — — amel.: spig.
— — **side**: ars. cact. calc. gels. mag-s. puls. spig.
— — — amel.: merc-i-r.
— — **temple**: aur-m. coca. lil-t. nat-ar. nit-ac. ped. sulph.
— — **vertex**: bar-ac. caus. cimic. nicc. nitr. podo. sep. sulph.
— — — amel.: ol-an.
**Morning to noon**: conv-d. ipec. nat-m. phos. sep.
— from 9 to 9 P. M.: mur-ac.
— to 5 P. M.: mang.
— to 10 P. M.: phys.
**Forenoon**: æth. alum. ant-c. bry. calc. canth. carbn-s. chin-s. cimic. cinnb. clem. cob. cocc. cocc-c. cop. cupr-ar. gamb. gen. ham. hydrs. indg. jac. jabor. k-ca. kalm. lach. lachn. lact. merc-i-fl. nat-m. nicc. phel. phyt. polyg. ptel. rhod. rhus. rumx. sep. sulph. tong. trom.
— **brain**: fluor-ac. indg. ran-b.
— **forehead**: ars-i. brom. bry. calc-s. chin. clem. cocc. coloc. con. dig. euphr. fluor-ac. gamb. gels. ign. k-ca. lach. lyc. mag-s. merc-i-r. myric. nat-ars. peti. plect. rhus. sars. selen. seneg. sep. *sulph.* ustil. znc.
— — amel. in: indm. lact.
— **occiput**: agar. all-c. alum. cob. dios. gels. lact. lyc. nat-c. phys. phyt. psor. spong. sulph.
— **side**: alum. cact. carb-an. castor. euphr. fluor-ac. hydrs. indg. jug-c. kalm. lach. nat-m. nx-j. peti. plb. sars. stront. verat.
— **temple**: alum. ars. asar. *cham.* clem. cob. dios. fago. gen. hipp. hydr. indg. *jug-c.* k-ca. lach. lil-t. lycps. mag-s. nat-ars. peti. phyt. podo. rhus. seneg. sulph.
— **vertex**: alum. bar-ac. bov. bry. calc. fluor-ac. gamb. glon. k-cy. mag-s. nat-ars. nicc. nx-m. pic-ac. rhus. sulph.
**Noon**: arg. ars. bov. calc-p. carb-v. cham. chel. cic. cob. con. graph. gymn. hydrph. ign. indg. jabor. k-bi.

kalm. lyc. lycps. mag-c. mang. merc. mur-ac. nat-c. nitr. phos. puls. rhus. spong. sulph. znc. zing.
— — to evening: sil.
— **forehead**: chel. dirc. fago. ign. puls-n. spire. *sulph.* verat. znc.
— **side**: calc-p.
— **temple**: dios. fago. pallad. ptel. sulph.
— **vertex**: *puls-n. sulph.* thu.
**Afternoon**: acon. æth. agar. alum. ambr. am-c. am-m. anac. ant-t. arn. ars. asar. aur. bad. bar. bell. berb. bov. bry. bufo. calad. calc. calc-p. canth. carb-an. carb-v. caus. chel. chin. chin-s. cic. cimic. cob. coca. cocc. colch. coloc. con. conv-d. creos. cund. cyc. dig. dios. dros. equ. euphr. fago. ferr. form. gamb. gels. gen. glon. graph. grat. ham. hell. hydrph. iber. ign. indg. indm. iod. k-ca. kalm. lac-can. lach. lact. laur. lepi. linu. *lyc.* lycps. mag-c. mag-m. mag-s. mang. merc-i-r. mezer. mur-ac. nat-c. nat-m. nit-ac. nitr. nx-m. nx-v. op. pallad. ped. petr. phos. ph-ac. phyt. pic-ac. plat. plb. polyg. ptel. puls. ran-b. rhus-r. ruta. sabin. sars. secale. selen. seneg. sep. sil. spong. stram. stront. sulph. sul-ac. tabac. tellur. valer. znc.
— — see also under Evening.
— — **amel.** in: ipec. ol-an.
— **brain**: bar-ac. hell. iris. lact. mag-s. merc-i-fl. uran-n.
— **forehead**: ailan. *aloe.* alum. ambr. anac. arg-n. bad. borax. bov. bry. bufo. calc-s. cann-i. castor. caus. chin. chin-s. cic. cimic. coca. colch. con. creos. cyc. dios. dirc. fago. form. gels. glon. graph. hipp. hydrph. ign. indm. ir-fœ. jabor. k-ca. k-cy. lact. laur. lil-t. lyc. mag-s. mang. merc-i-r. mur-ac. myric. naja. nat-ar. nat-m. nit-ac. nitr op. ped. peti. phos. ph-ac. pip-m. plect. puls. ran-b. rhus-r. sang. senec. serp. sil. sol-t-æ. stront. sulph. tabac. tarent. valer.
— — amel. in: bar-ac. ferr.
— **occiput**: æth. agar. ang. bov. canth. castor. chel. chin-s. cimic. clem. coca. dios. dirc. fago. hydrs. indm. iod. iris. mang. nitr. ol-an. osm. ph-ac rhus. rhus-r. rumx. sars. sep. sulph.
— **side**: æth. alum. bry. canth. castor. chin-s. coca. colch. ferr. graph. indg. lach. laur. mag-s. merc-i-r. nat-m. nicc. nit-ac. nx-m. ol-an. *sep.* valer. znc.
— **temple**: aloe. alum. bell. bov. bry. canth. caus. chin-s. coca. cod. coloc. corn. dios. dirc. dulc. equ. fago. gamb. grat. guai. hipp. iber. iod. k-bi. laur. lyc. mag-s. myric. nat-ar. nit-ac. ol-an. peti. plat. ptel. rumx. sang. sil. stront. sulph. zing.
— **vertex**: alumn. alum. ambr. ars. bufo. calc-s. carb-v. cimic. crotal. graph. helon. hura. hydrph. hyper. indm. ir-fœ. lac-ac. lyc. mang. merc-i-r. mur-ac. nat-ar. nit-ac. nitr. osm. phel. phos. phys. sulph.
**Evening**: acon. agar. all-c. alum. ambr. am-c. anac. ang. ant-t. apis. arg. ars. aur. bad. bar. bell. borax. bov. brom. bry. calc. camph. canth. caps. carb-an. carb-v. caus. cedr. cham. chel. chin. cic. cimic. cina. clem. cob. cocc. cocc-c. colch. coloc. croc. croton. cyc. cupr. cupr-ar. dig. dios. dulc. elat. eugen. euphr. ferr. form. glon. graph. hell. hepar. hipp. hydrs. hydrph. hyper. indg. indm. iris. jug-c. k-bi. k-ca. k-clc. kalm. lach. lachn. lac-ac. laur. led. lept. lil-t. lobel. lyc. lycps. mac. mag-c. mag-m. mag-s. mang. marum. men. meph. merc. merc-i-fl. merc-i-r. mezer. mosch. murx. mur-ac. nat-c. nat-m. nit-ac. nitr. nx-j. nx-v. par. pau-p. petr. phos. ph-ac. phys. plan. plat. plect. plb. psor. puls. ran-b. ratan. rhod. rhus. ruta. sabad. sabin. sang. sars. selen. seneg. sep. sil. spig. spira. stann. staph. stram. stront. sulph. sul-ac. tellur. tereb. therid. thu. trif-p. valer. wies. znc.
— — **aggravated**: all-c. asaf. bell. cham. chin-s. cist. cyc. elaps. ferr. hipp. k-iod. kalm. mag-c. mezer. nat-m. par. puls. sep. tilia.
— — **better in**: *bry.* ham. k-bi. lach. mang. nat-ar. *nat-m.* phys. pic-ac. sang. spig. tereb.
— — — see also under Morning.
— — 1 P. M.: ailan. coca. lyc. phys. pic-ac. ptel.
— — 1 to 3 P. M.: chin-s. plan.
— — 1 to 5 P. M.: lac-ac. mag-c.
— — 1 to 10 P. M.: mag-c. plat. spig.
— — 2 P. M.: grat. hydrph. laur. phys. ptel.
— — 2 P. M. to 7 A. M.: bad.
— — 2 P. M. till late in the evening: bad.

— — 3 P. M.: fago. guai. hura. hydrph. iber. lycps. nat-ar. sep. sil. thu. verat-v.
— — 3 to 4 P. M.: brom. clem.
— — 3 to 9 P. M.: arn. hydrph. nat-ar. tarent.
— — 4 P. M.: arg-n. chin-s. dios. helon. meni. nat-m. phys. pic-ac. stryc. *sulph.* verat-v.
— — 4 to 8 P. M.: hell. lyc.
— — 4 P. M. to 3 A. M.: bell.
— — 5 P. M.: bufo. equ. helon. nat-m. pæon. pic-ac. ptel. sulph.
— — 5 to 6 P. M.: chin-s. lil-t.
— — 5 to 9 P. M.: plat.
— — 6 P. M.: cob. gels. nat-s. pæon. ptel.
— — 7 P. M.: chin-s. cocc. elaps. lyc. mag-c. nat-m. rhod. sep. *sulph.* verat-v.
— — 8 P. M. gymn. lac-ac. merc-i-r. phys. sol-n. stryc. sulph.
— — 8 to 9 P. M.: helon. indg.
— — 9 P. M.: coca. dios. eupi. gels. hydrph. osm. pic-ac. ptel.
— — 10 P. M.: dios. ham. laur. myric. phys.
— — 11 P. M.: dios. indg. merc-i-r. pip-m. stram. valer.
— — twilight, at: ang. puls.
— **brain**: agn. all-c. nat-m. par. phos. ran-b. znc.
— **forehead**: acon. agar. alumn. alum. anac. ang. ant-t. aran. arg. arg-n. ars. arum-t. bad. bapt. bar. bism. borax. bov. bry. cact. calc-s. camph. castor. caus. chel. chin. chin-s. cimic. cina. cinnb. cocc. crotal. *cyc.* dig. dios. dulc. erec. erig. ery-m. fago. ferr. fluor-ac. graph. ham. hell. hipp. hura. hydrph. iber. indg. indm. iod. iris. ir-fœ. k-ca. k-iod. kalm. lach. lac-ac. lepi. lil-t. lyc. lycper. mag-c. mag-m. mag-s. merc. merc-i-fl. merc-i-r. myric. nat-m. nit-ac. nitr. nuph. nx-j. ol-an. osm. pæon. ped. peti. phos. ph-ac. phys. pic-ac. plat. plect. plb. podo. psor. *puls.* ran-b. ran-sc. ratan. rhus-r. rumx. sars. selen. seneg. serp. sil. sin-n. staph. *sulph.* sul-ac. thu. uran. ustil. valer. znc.
— — **better** in: chin. clem. coca. k-bi. naja. op.
— **occiput**: alum. ambr. bar. bell. bov. canth. carb-an. chin-s. colch. dios. form. gels. graph. hyper. indg. jabor. k-bro. k-clc. lobel. lyc. mag-c. mezer. mur-ac. nit-ac. nitr. ol-an. op. ptel. ran-b. ran-s. rhus-r. seneg. sep. stann. staph. stront. sulph. thu. uran. znc.
— — **better** in: coca. serp.
— **side**: aloe. arg. bar. calc-s. canth. caus. chin. dios. elaps. fluor-ac. graph. ham. hydrph. indg. indm. k-ca. lyc. mag-c. mag-m. merc-i-r. mezer. nicc. nitr. nx-v. pallad. phos. puls. sep. sil. spig. sulph. tabac. tong. znc. zing.
— **temple**: acon. aloe. alum. am-c. ang. apis. aran. calc-s. camph. castor. caus. chin. cinnb. colch. cop. creos. crotal. dig. dios. equ. fluor-ac. hydrs. hyper. inu. jac. k-ca. k-iod. lach. lac-ac. lepi. lith. mag-m. mezer. nat-m. nit-ac. nitr. nx-m. nx-v. ph-ac. psor. ran-b. rhus-r. sep. stram. stront. sulph. sul-ac. tabac. tarent. thu. zing.
— **vertex**: acon. ambr. apis. borax. canth. carb-an. cimic. conv-d. crotal. cyc. dulc. fago. form. gent. glon. hepar. hyper. k-ca. k-iod. lach. lith. lyc. merc. mur-ac. nit-ac. ol-an. petr. ran-b. rhus. sep. sil. stront. *sulph.* thu. znc.

**Evening in bed**: ars. carb-v. cyc. hipp. laur. lyc. mag-c. phos. *puls.* sep. sulph. znc.
— — — better: mag-c. sulph.
— **forehead**: fluor-ac. mag-s. sep.
— **occiput**: dulc. nitr. sarr.
— **side**: arg. con. plat.
— **temple**: chel. glon. ol-an. ph-ac. rhus.
— **vertex**: carb-v. stann.

**Night**: alum. ambr. am-c. am-m. anac. ang. *ant-t.* arn. ars. arum-t. aster. bell. berb. borax. bov. bufo. cact. calc. camph. canth. carb-an. carb-v. caus. cedr. cham. *chin.* chin-s. cic. cist. con. colch. creos. cupr-ar. cyc. dig. dulc. elaps. eugen. glon. graph. grat. guai. ham. *hepar.* hydr-ac. hyos. ign. indm. k-ca. lach. lact. lam. laur. led. lobel. *lyc. mag-c.* mag-m. meni. *merc.* mezer. mill. nat-c. nat-m. nit-ac. nitr. nx-m. nx-v. op. par. pau-p. phos. ph-ac. plat. puls. raph. rhus. rhus-r. sars. sep. sil. spig. stront. *sulph.* tarent. thu. verat. znc.
— — compare with Evening.
— — **aggravated** at: ars. bov. calc. clem. puls. sil. tarent. thu.
— — **better** at: bufo. ham. sol-t-æ. spire.
— **forehead**: anac. arg-n. ars. camph.

caus. chin-s. cinnb. croc. crotal. cyc. fago. ham. hepar. hura. k-ca. lachn. lac-ac. lyc. mag-s. merc-i-r. naja. pip-m. ptel. puls-n. raph. sang. sil. sin-n. spig. tarent. tilia.
— — **better**: clem. phys.
— **occiput**: borax. carb-v. clem. hipp. lyc. nitr. sep. stront. *sulph.*
— — **better** at: bov. glon. graph.
— **side**: cact. graph. mag-c. nat-m. nicc. nitr. ol-an. tarent.
— — better: mag-c.
— **temple**: arn. ars. arum-t. bry. cact. cop. dig. ferr. grat. k-ca. lyc. mag-s. rhus-r. sang. tarent.
— **vertex**: acon. agar. aster. carb-an. ferr. glon. hipp. ir-fœ. laur. lyc. nitr. ol-an. ratan. sulph.
— — **better**: mag-c.
— — — on going to sleep: phyt.
**Night in bed**: aloe. alumn. fago. hipp. hyper. merc-i-fl. sulph.
— — — see Bed, and compare Evening in bed.
**Night on waking**: alumn. ambr. ant-t. aster. bufo. canth. cinnb. coloc. ferr. gels. gins. glon. hyper. lac-ac. mang. merc-i-fl. ment-pi. mezer. ph-ac. plect. rumx.
— — — see also Waking.
**Midnight**, before: am-m. anac. caus. chin. dulc. lach. puls. rhus. sep.
— — see hours given under Evening.
— **about**: all-s. arn. ars. elaps. hepar. k-ca. mag-s. myric. plat. puls. sep.
— **after**: ars. carb-an. cham. ferr. hepar. ign. k-ca. ph-ac. rhus. sep. sil. spig.
— — 1 A. M.: pallad.
— — 3 A. M.: thu.
— from, till noon: hepar.
**Air**, **open**: alum. ang. arg-n. bell. bov. bry. cadm. *calc.* calc-p. caus. cedr. chel. chin. cina. cocc. coff. con. eup-per. euphr. ferr. glon. grat. hepar. hipp. ign. iod. ipec. k-ca. kalm. lach. laur. lyc. mang. men. mezer. mur-ac. nat-m. *nx-v.* petr. phos. ran-b. rhus. spig. staph. sulph. valer. znc.
— — — see also under Walking in open air, and compare with Weather.
— — — **aggravated**: arg-n. bell. calc-p. eup-per. grat. kalm. lil-t. men. mezer. spig. sulph.
— — — **better**: acon. all-c. alum. am-c. ang. ant-c. ant-t. aran. ars. asar. aur. bell. berb. bov. calc. camph. cann-s. caus. cimic. clem. cob. coff. coloc. croc. diad. dulc. fago. ferr. ferr-i. glon. grat. ham. hell. hydr-ac. hydrph. hyos. iod. jatr. k-ca. k-iod. lach. laur. lyc. mag-m. mag-s. mang. men. mezer. mosch. nat-c. nat-m. nicc. nitr. olnd. op. petr. phel. phos. plat. puls. ran-b. rhod. sars. seneg. sep. stann. sulph. sul-ac. tabac. tarent. thu. viol-tr. znc.
— — — headaches that come on in-doors are better out-doors and *vice versa;* mang. ran-b.
— — **forehead**: bell. calc. chel. euphr. k-bi. lachn. mang. nx-v. plect. rumx. staph.
— — — **aggravated**: agar. chel. sil.
— — — **better**: acon. alum. ang. arg-m. aur. aur-m. berb. calc. camph. carb-an. castor. cimic. colch. coloc. coral. croton. euphr. ferr. ferr-i. ham. hell. hydr-ac. jac. k-bi. lach. mag-s. nuph. nx-j. sars. sep. sulph. tabac. tarax. viol-tr.
— — **occiput**: cob. hydr-ac. iod. lobel.
— — — better: carb-an. glon. hydrs. k-ca. mag-m. mag-s.
— — **side**: fluor-ac.
— — — better: am-c. carb-an. fago. grat. mang. phos. ratan. *sep.* sulph.
— — **temple**: aur. coloc. equ. hyos. jac. k-bi. mang. naja. ol-an.
— — — better: asar. atro. camph. castor. coloc. com. croton. glon. hell. hydrs. hyos. jatr. lith. nuph. olnd. phos.
— — **vertex**: ferr. iris. sulph.
— — — better: carb-an. ferr. gamb. glon. indm. nitr. ratan.
**Air**, **cold**, from: aur. bov. calc. camph. carb-an. caus. cocc. coff. ferr. grat. ign. k-bi. nat-m. nx-v. plat. rhod. rhus. ruta. sil.
— — **ameliorates**: aloe. hydrph. sin-n.
— **draught** of, from: *acon.* ars. *bell.* benz-ac. cadm. *calc.* caps. chin. coloc. hepar. k-ca. nitr. nx-m. *nx-v.* phos. selen. *sil.* sulph. valer. verb.
— — of cold, damp: cimic. dulc. mosch.
— — of cold, dry: acon.
— **vaults**, cellars, churches, etc.: *ars.* bry. carb-an. *puls. sep.* stram.
— **warm**, from: acon. am-c. arn. bar. bell. bry. carb-v. caps. euph. ign. iod. ipec. laur. led. mosch. puls. seneg. sep. spong.

**Acids**, from : bell. morph-acet. *selen.*
— ameliorate : tong.
**Alcohol**, from abuse of : acet-ac. *ant-c.*
— smell of : sol-t-æ.
— see also Beer, Spirituous liquors, Wine, etc.
**Angry**, when getting : dulc.
— after being : *bry.* *cham.* coloc. ign. lyc. mag-c. mezer. nat-m. nx-v. petr. phos. plat. ran-b. rhus. staph.
**Animal** fluids, from loss of : ars. *calc.* *carb-v.* *chin.* cina. cocc. con. k-ca. lach. merc. *nat-m.* *nx-v.* phos. *ph-ac.* *puls.* *sep.* *sil.* *staph.* *sulph.* verat.
— — after profuse uterine hemorrhages : *glon.*
— — see also Sexual excess.
**Arms**, from moving : caus. nat-s. ptel. rhus. spong.
**Arthritic** : acon. am-c. arn. ars. *asar.* aur. *bell.* benz-ac. bry. camph. *caps.* caus. cham. chin. *cic.* cina. *coloc.* con. *eugen.* graph. guai. hyos. ign. ipec. mag-c. mang. mosch. nat-c. *nat-m.* *nit-ac.* *nx-v.* petr. phos. plat. puls. rhus. sabin. sep. spig. *verat.* znc.
**Ascending** steps, etc. : alum. ant-c. arn. aster. bell. cadm. *calc.* cimic. crotal. cupr. ferr. gels. glon. hydrs. ign. kalm. lach. lobel. lyc. men. mosch. nat-ar. nx-v. par. ph-ac. ptel. rhus. sil. spong. staph. sulph.
— **forehead** : ant-c. arn. cimic. ign. men. sulph.
— **occiput** : bell. carls. pic-ac.
— **side** : hydrs.
— **temple** : glon. kalm. sulph.
— **vertex** : ant-c. cimic. ferr. lobel. men.
**Attention**, from too eager : anac. nx-v. sabad.
— — see also Mental exertion.
— being called to pain, aggr. : cham. chin. ferr-p. helon. ign.
— — see also Thinking of.
**Awake**, when trying to keep : phys.
**Back**, better from pressing up against something hard : sang.
**Bandaging**. See Binding.
**Bathing**, after : *ant-c.* bell. calc. canth. caus. creos. *nit-ac.* *rhus.* puls. sep.
— see also Washing, Water, etc.
— ameliorates : lac-ac.
— cold : bell. nit-ac. phos. *rhus.* sep.
— sea : ars. *rhus.* sep.
**Bed**, on going to : alum. ars. lyc. mag-m. puls. sabad. sep. sulph. znc.
— heat of, aggr. : lyc.
— must leave the : coloc.
— better in : colch. rhus. sep.
— see also under Morning, Evening and Night.
**Beer**, from drinking : all-c. bell. calc-cau. *cocc.* coloc. ferr. merc. *rhus.* verat.
— and bread, aggr. from : croton.
**Bending**. See Head, bending, and under Stooping.
**Binding** or bandaging head, from : calc. cham. lach. rhus. thu.
— see Pressure, etc.
— ameliorates : *arg-n.* bell. bry. *calc.* hepar. mag-m. nx-v. psor. puls. rhod. *sil.*
— — up hair, amel. : nitr.
**Blows**, from : *arn.* hyper. nat-s. See Injuries.
**Boat**, from riding in a : *cocc.* colch. ferr.
**Brain** fag, from chronic : calc.
— see also Mental exertion.
**Bread**, from eating : manc. zing.
— and beer, aggr. : croton.
**Breakfast**, before : cimic. indg. rumx.
— **after** : bufo. cham. hydrs. indg. *lyc.* naja. nat-m. nit-ac. nx-m. *nx-v.* par. petr. phos. ph-ac. plb. sul-ac.
— — better : am-m. arum-t. bov. canth. carb-ac. con. croc. eup-per. nat-p.
**Breathing**, deeply, on : anac. ratan.
— when holding the breath : agar.
**Carriage**. See Driving.
**Catarrhal** : acon. æsc-h. all-c. alum. ambr. am-m. ars. bell. bry. carb-v. caul. cham. chin. cic. cimic. cina. dulc. euphr. gels. gymn. hell. hepar. hydrs. ign. k-bi. k-ca. k-iod. lach. laur. lyc. merc. mezer. nat-ar. nat-m. *nx-v.* puls. ran-b. sabad. samb. sang. staph. stict. still. *sulph.*
**Chagrin**. See Anger.
**Chewing**, when : k-ca. olnd. phos. sulph.
— — **temples** : am-c. am-m. k-ca.
**Chill**, before the : æsc-h. ars. bell. *bry.* calc. carb-v. cedr. chin. corn-f. elat. ipec. lach. nat-c. *nat-m.* nitr. plant. puls. rhus. spong. *thu.*
— **during** the : acon. am-c. anac. ang. ant-t. *aran.* arg-n. arn. ars. bapt. *bell.* berb. borax. bov. bry. cact. calc. camph. caps. carb-an. carb-v. cham. chin. chin-s. cimic. cina. coca. coloc. coral. creos. crotal. daph. dros. dulc. elat. eup-per. *eup-pur.* eupi. ferr. gels. graph. hell. hepar. hipp. ign. ipec. k-ca. lach. lact. led. mag-c. mang.

mezer. NAT-M. *nx-v.* petr. phos. podo. puls. rhod. rhus. ruta. sang. seneg. SEP. spig. spong. stram. *sulph.* tarax. thu. verat.
— **after** the: ant-t. cedr. dros. NAT-M.
**Choraic** persons, in: *agar.*
**Chronic**: am-c. con. sulph.
— of old people: iod.
— see also Constant.
**Climaxis**, during the: carb-v. croc. glon. lach. sang. therid. ustil.
**Coffee**, from abuse of: acet-ac. arg-n. arn. arum-t. bell. calc-p. caus. *cham.* cocc. form. glon. hepar. *ign.* lach. lyc. merc. mill. nitr. *nx-v.* puls.
— **relieves**: cann-i. chin. coloc. glon. hyos. tilia.
— from smell of: lach.
**Coition**. See under Sexual.
**Cold applications** relieve: acon. aloe. ant-t. ars. asar. aur-m. bell. bry. calc-p. cham. chin-s. cinnb. cyc. euph. euphr. ferr. glon. indm. lac-can. merc-c. myric. phos. plant. seneg. spig. znc.
— **becoming**, from: acon. agar. ant-c. ars. bry. cadm. carb-an. carb-v. clem. colch. grat. lach. mosch. nit-ac. petr. phos. puls. stram. stront. sul-ac. verb.
— — on head getting cold: *aur. bell.* hepar. hyos. k-ca. nx-v. puls. *sep. sil.*
— — on sudden change to cold: carb-v. dulc.
— **getting a cold**, from: *acon.* ant-c. arn. *bell. bry.* calc. carb-v. caus. *cham.* chin. coff. coloc. con. dulc. graph. hepar. hyos. *k-ca.* lach. merc. nat-m. nit-ac. *nx-v.* petr. *phos. puls.* rhus. samb. sep. *sil.* sulph. verat.
— **suppressed** cold, from having a: *acon.* am-c. ars. bell. bry. *calc.* carb-v. cham. chin. cina. lach. lyc. *nx-v.* puls. sil. sep.
**Company** or crowd, when in: mag-c. plat.
**Compress**. See under Pressure.
**Concussion**, from: arn. bell. cocc. ferr-p. hepar. phos.
— Compare with Jar, etc.
**Constant**, continued: arg. cann-s. cupr. dulc. hydrs. indg. lobel. lept. nat-m. phos. rhus-r. sep. still. tereb.
— fixed, lasts for weeks, months, even years, with rare intermissions: tereb.
— for two or three days: croc.
**Constipated**, when: aloe. alum. crotal. lach. nat-m. nx-v. petr.
— compare with Stool.
**Contradiction**, after: lyc. mag-c. nat-m. petr. phos. rhus.
**Copper**, abuse of: hepar.
**Coryza**, from suppressed: chin. *k-bi.* k-ca.
— see also Cold, suppressed.
— during. See under Concomitants.
**Coughing**, on: acon. alum. ambr. am-c. anac. ang. ant-t. apis. *arn.* ars. aur. bad. BELL. BRY. cact. calc. *caps.* carb-v. caus. chel. chin. CON. ferr. hepar. hydrs. hyos. ign. ipec. iris. k-bi. k-ca. lach. led. lyc. mag-s. mang. *merc.* mezer. NAT-M. nit-ac. nitr. *nx-v.* œna. ol-an. petr. PHOS. ph-ac. *puls.* rhus. rumx. ruta. *sabad.* sang. sars. sep. spig. *squil. stann.* SULPH. sul-ac. tarent. tril. ziz.
— — cough, alternating with headache: lach.
— **forehead**: acon. ant-t. arn. *asc-t.* brom. chel. coca. creos. ferr. hepar. hyos. k-bi. mosch. nat-m. phos. ruta. seneg. spong. staph. sulph. verb.
— — pain in, relieved by coughing: arg.
— **occiput**: alum. anac. carb-an. *ferr.* mag-c. merc. mosch. sep. sulph.
— **side**: apis. aur. cimic. dirc. mang. vibur.
— **temple**: alum. ambr. ant-t. caus. cina. coca. creos. k-ca. lyc. puls. rhus. tarent. taxus. verb.
— **vertex**: alum. *anac.* con. cupr. sabad. squil. sulph.
**Covering** head, from: calc. carb-v. chin. glon. laur. *led.* sulph. valer.
— — **relieves**: aur. hepar. mag-m. mur-ac. nx-v. psor. *sil.*
— — wears a fur cap even in hot weather: psor.
— — see also under Heat, Hat, etc.
— **uncovering** relieves: croton. glon. lobel. lyc. nitr. sulph. zing.
**Damp**. See under Air, Weather, etc.
— houses, living in, from: *ars.* calc. *carb-v.* phys. puls. rhod. *rhus.* sil. *verat.*
**Darkness**, relieves: acon. brom. hipp. sang. sep. sil.
— compare with Light, aggravates.
**Debauch**, after a: bry. carb-v. chin. cocc. creos. ipec. laur. led. nx-v. puls. sabin. stram.
**Dentition**, during: *acon.* bell. *calc. cham.*

hepar. hyos. ign. merc. nit-ac. rhus. sil.
**Descending**, on: ferr. men. merc-i-fl.
**Diarrhœa.** See under Stool.
— compare with same under Concomitants.
**Dinner**, before: indg. nx-v.
— from delayed: cact. cist.
— **after**: am-c. aran-s. bell. calc-p. castor. chin-s. cimic. con. dios. gins. gen. glon. hyper. jug-r. k-bi. lob-s. mag-m. merc-i-fl. nat-m. nat-p. nitr. nx-v. phel. phos. phyt. raph. sulph. thu. valer. znc.
— **relieved** after: arg-n. arum-t. phos. ptel.
**Dreams**, after unpleasant: cob.
**Drinking**, from: acon. cimx. cocc. merc.
— **cold** drinks, from: con.
— — — relieve: alum. k-ca.
— **milk**, from: brom. phys.
— **quickly**, after: nat-m.
— **tea**, from abuse of: carb-ac. selen. thu.
**Driving**, or motion of carriage, from: ars. *cocc.* colch. ferr. graph. hepar. ign. iod. k-ca. lach. meph. naja. nit-ac. nx-m. *sep.* sil. sulph.
— — too long: iod.
— — **relieves**: brom. merc. nitr.
— **forehead**: acon. glon. lyc. nx-m.
— **temple**: lith. lyc.
— **vertex**: lyc.
**Drugs**, after abuse of: nx-v.
**Eating, before**: cann-s. carb-an. nx-v. ran-b. sabad. sil.
— **when** eating: am-c. chel. cocc. con. dulc. gels. graph. mag-m. manc. nat-m. nit-ac. nx-v. puls. ran-b. rhus. sabin. secale. sul-ac. tabac. verb. znc.
— — **relieved**: chel. phel. sin-n. sulph. znc.
— — better while eating, but worse after: *anac.* lith.
**Eating, after**; after meals, etc.: agar. alum. ambr. am-c. *anac.* ant-c. arn. ars. bar. bell. bov. *bry.* bufo. *calc.* calc-p. canth. caps. carb-an. *carb-v.* castor. caus. cham. chel. chin. chin-s. cina. cinnb. cocc. coff. croton. dios. evon. ferr. gels. gen. glon. graph. grat. hyos. ign. indm. k-ca. lach. laur. lobel. lyc. mag-c. mag-m. men. merc. merc-i-fl. merc-s. mur-ac. nat-c. nat-m. nat-s. nit-ac. nitr. nx-j. nx-m. *nx-v.* pæon. petr. phel. phos. ph-ac. plat. prun. puls. ran-b. rhus. ruta. sars. seneg. sep. sil. staph. sulph. valer. verat. znc.
— — **relieved**, after: arg-n. arum-t. carb-ac. caus. cist. coca. con. gels. gen. indm. lachn. lyc. petr. phos. phyt. scut. sep. tellur. tong.
— **brain**: canth. indg. ran-b.
— **forehead**: alum. am-c. aran. bov. brom. bry. calc-s. calen. carb-v. cham. chel. chin. chin-s. clem. colch. con. graph. hydrs. inu. k-bi. k-bro. k-ca. lyc. mag-c. nat-s. nitr. op. phos. phyt. plat. sars. sulph. tabac. valer. znc.
— — **relieved**: carb-an. chel. cist. gen. phyt. psor.
— **occiput**: agar. alum. canth. carb-v. dios. gels. mill. nat-m. ol-an. pip-m.
— **sides**: bar. bell. calc-s. cocc-c. form. ham. k-ca. lach. mag-c. nx-v. pæon. phos. znc.
— — relieved: calc-p. colch. form. nat-m. tong.
— **temple**: alum. aran. canth. castor. clem. con. dios. hydrs. indg. k-bi. mag-c. nitr. ol-an. phos. znc.
— **vertex**: bad. calc-s. castor. dirc. inu. k-bi. lyc. mag-c. nat-c. phel. rhus. sulph. tabac.
**Effort.** See Exertion.
**Emotions.** See Excitement.
**Epileptic** attacks, after: cina. cupr.
**Eructations** relieve: gent-c. lach. mac.
**Eruption**, suppressed, after: *ant-c.* bry. lyc. nx-m. sulph.
**Excitement**, after: acon. arg. bry. cact. cham. chin. chin-s. cocc. coff. con. creos. cyc. gels. ign. nat-m. nx-v. op. par. ph-ac. rhus. scut. staph. tanac.
— depressing or sad, after: cocc. ign. nx-v. op. staph.
**Exertion**, of body, etc.: acet-ac. anac. berb. cact. calc. calc-p. gins. glon. lact. mezer. nat-c. nat-m. rhus-r. spong. zing.
— **relieves**: agar. mag-m. merc-i-fl. rhus-g.
— compare with Motion.
**Eyes, on moving** the: acon. agn. am-c. arn. bar. bell. *bry. caps.* chel. *chin.* chin-s. coloc. con. croton. cupr. dig. dros. hepar. ign. indm. mag-s. mur-ac. nat-m. nx-v. *op.* puls. rhus. sep. *spig.* sulph. valer.
— — **forehead**: bad. chel. chin-s. dros. gels. hepar. ign. mur-ac. nx-j. puls. rhus. spig. valer.

— — — moving the eyelids: bry. coloc.
— — **temple**: bad. chin. sulph.
— — **vertex**: sep.
— **opening**, on: *bry.* chin. coff. con. gent. ign. mag-c. nx-v. rhus. spig. sulph.
— — relieves: chin.
— — morning, on first: *bry.*
— **closing** the, on: all-c. bry. nx-v. sabin.
— — relieves: acon. calc. con. hell. ipec. iod. ipec. spig. sulph.
— **turning** the, on: dig. rhus. sep.
— — up the, on: bell. caps.
— see also under Looking.
**Face**, on moving the: apis.
**Fainting**, after: mosch.
**Fall**, after a: arn. hyper. rhus.
**Fasting**, when: cist. elaps. indm. iod. nx-v. ptel. ran-b. *sil.* sulph. uran-n.
— compare with Relieved after eating.
**Fat** food, from: *carb-v.* colch. cyc. ign. nat-c. nat-m. *puls.* sep. thu.
**Fatigue**, from: calc. sil.
**Feet, cold**, from getting: cham. k-ca. phos. puls. sil.
— **wet**, from getting: gels. phos. *puls.* rhus. sep. *sil.*
**Fever, before** the: bry. chin. puls. rhus. spong.
— **during** the: acon. æsc-h. agar. am-c. *ang.* ant-t. ARN. *ars.* asaf. BELL. berb. borax. bry. cact. calc. camph. caps. carb-v. CHIN. chin-s. cina. coloc. corn-f. croton. cupr. dros. dulc. elat. EUP-PER. graph. *hepar.* hipp. hyos. *ign.* k-bi. k-ca. lach. lobel. lyc. NAT-M. *nx-v.* op. plant. *podo.* *puls.* *rhus.* ruta. *sabad.* sep. SIL. spig. sulph. *thu.* valer. verat.
— **after** the: ars. calc. *carb-v.* EUP-PER. NAT-M.
**Flatulency**, as if from: carb-v. chin-s. mag-c. nit-ac. sulph.
**Flatus**, emission of, relieves: æth. cic.
**Food**. See Eating, or Special kind.
**Fire**. See Heat.
**Fright**, after: acon. calc. cupr. hipp. hyos. *op.* ph-ac. plat. puls. samb.
**Frowning**, from: ars. mang. nat-m.
— relieves: calc-cau.
**Gaslight**, from working under: glon.
**Gastric**: acet-ac. acon. æsc-h. ailan. alum. am-c. anac. *ant-c.* apis. arn. ars. asar. atro. bell. berb. bism. bry. calc. calc-p. caps. caul. caus. carb-v. cham. cic. cina. cocc. coff. eup-per. form. gamb. gels. glon. hydrs. ign. indg. *iris.* k-bi. k-ca. lach. lept. lyc. naja. *nx-v.* op. par. phos. phyt. plat. *puls.* rubin. sang. sep. sil. stict. *sulph.* *tabac.* tarent. verat.
— compare with Concomitants.
**Girls**, in young: acon. bell. calc-p. nat-m. ph-ac. puls.
**Grief**, from: ign. ph-ac. staph.
**Hair**, after cutting: bell. glon.
— from combing: glon. merc-i-fl.
— — — amel.: form.
— from touching the: agar.
— **tying** up, relieves: nitr.
**Hand**, from pressure of: marum.
— cold, relieves: calc.
— pressure of, relieves: aran-d. cinnb. con. dros. glon. men. merc. olnd. par. rhus. sabad. spig.
— — see also under Pressure.
**Hat**, from pressure of: calc-p. carb-an. carb-v. croton. glon. hepar. laur. merc. sulph. valer.
**Head, bending backward**, when: anac. aur. bell. clem. cupr. dig. elaps. glon. lyc. mang. osm. spig. stann. valer.
— — — — relieves: apis. glon. ph-ac. thu.
— — — **forehead**: chin. stann.
— — — — relieves: bell. thu. verat.
— — — **occiput**: anac. colch. osm.
— — — — relieves: æth. bar-ac. chin. murx. ph-ac. raph. rhus. spig.
— — — **temple**: anac. thu.
— — **forward**. See Stooping.
— **holding erect**, when: bar.
— — must hold head and eyes down: apis.
— **leaning** against something, when: ang. bell. cyc. nat-m.
— — — relieves: anac. aral. arn. bell. brom. cann-s. con. diad. dros. gels. gymn. k-bi. men. merc. nx-v. rhod. sabad. sabin. sang. seneg. spig. sulph.
— **lies** with head low: absin. arn. cadm. *nx-v.* phys. spong.
— — — — high: caps. gels. nat-m. puls. spig. spong. stront.
— **moving** head, when: acon. am-c. arn. ars. bar. bell. berb. bry. calc. camph. cann-s. canth. caps. caus. chin. chin-s. *cimic.* cic. clem. cocc. cocc-c. colch. con. coral. corn. cupr. dros. euph. ferr-i. fluor-ac. gels. gen. gent. glon. graph. hell. hepar. ipec. k-ca. lach. lact. lyc. mag-c. mang.

mezer. mosch. nat-c. nat-m. nx-j. nx-m. nx-v. ph-ac. plat. puls. rhod. samb. sang. sars. secale. sep. sil. spig. spong. staph. sulph. therid. verat. vibur. viol-od.
— — see also Shaking.
— — **relieves**: cina. plan.
— **raising** head, when: ang. bar-ac. bov. cact. calc. caps. chin-s. coca. dros. ign. lach. seneg. spong. squil. sulph. tarax. verat. viol-tr.
— — **relieves**: ang. carb-v. ign. k-ca. mag-c.
— **resting** on arm, when: nat-m.
— — — relieves: dros. seneg. staph.
— — on hand, when: bell. chin.
— **shaking** head, when: acon. arn. ars. bar. bell. bry. caus. chin. coloc. con. glon. hepar. lact. lyc. mang. nat-m. nit-ac. nx-v. ph-ac. rhus. ruta. sep. spig. squil. stann. staph. sul-ac.
— — partially relieves: gels.
— **side**, bending head, to painful, when: mezer. tabac.
— — to one: chin. men.
— — — — relieves: men. puls.
— **turning** head, on: canth. clem. coloc. gen. glon. graph. hyos. lyc. nat-c. nat-m. nitr. phos. ph-ac. phys. pic-ac. spong.
— — **forehead**: canth. chin-s. cocc-c. gels. glon. nat-c. ph-ac. rhod.
— — — quickly: nat-c.
— — — to right: æth.
— **wrapping** up. See Covering.
**Heat**. See Fever.
— **relieves**: *aur.* bell. caps. caus. cinnb. cocc. colch. *k-ca.* k-iod. lach. *mag-m.* nx-m. nx-v. rhod. rhus. *sil.* staph. stront. sulph. sumb.
— — heat of hand: cinnb. iris.
— — hot applications: cinnb. colch. iris. k-iod. mag-m. sil.
**Heated**, from becoming: *acon.* aloe. am-c. *ant-c.* arg-n. arn. bar. bell. *bry.* calc. camph. caps. *carb-v.* con. dig. glon. grat. ign. ipec. lyc. nat-m. nx-m. op. ptel. sep. sil. staph. thu. znc.
— — — by a fire: *ant-c.* *bar.* bry. euph. merc. puls. *znc.*
— — — of bed: nx-m.
**House**. See Room, and under Damp.
**Hunger**, from. See Eating, Fasting, etc.
**Hypochondriacs**, in: *nx-v.* staph.
**Hysterical**: arn. asaf. bell. cann-s. caps. cham. cimic. cocc. coff. gels. hell. hepar. hyos. ign. iris. lach. lact. mag-c. mag-m. nit-ac. nx-v. phos. ph-ac. plat. rhus. ruta. scut. sep. stict. stram. tarent. valer. verat.
**Indoors**. See Room.
**Injuries**, mechanical, after: *arn.* calc. *cic.* con. dulc. hepar. *hyper.* lach. merc. nat-s. nit-ac. petr. phos. puls. rhus. staph. sulph. sul-ac.
**Inspiration**, during an: *anac.* carb-v.
**Intoxication**, after: *ant-c.* bell. *bry.* *carb-v.* cocc. coff. glon. laur. *nx-v.* *puls.* spong. stram. sulph. tarax.
— see also under Spirituous Liquors.
**Iron**, from abuse of: puls. znc.
**Jar**, from any: *bell.* *chin.* ferr-p. glon. hepar. ph-ac. sang. sil. spig. sulph. *therid.* vibur.
— see also Concussion.
**Jaw**, moving. See Chewing.
**Joy**, from excessive: coff. cyc. op. puls. scut.
**Labor**. See Exertion; also under Mental.
**Laughing**, from: iris. mang. phos. tong. znc. zing.
**Lemonade**, from: selen.
**Lie down**, must: con. croc. ferr-ac. gels. mag-m. ph-ac. rhus. stict.
— — see Lying relieves.
**Lifting**, from: ambr. *arn.* bar. bry. *calc.* cocc. graph. lyc. nat-c. nx-v. ph-ac. *rhus.* sil. sulph.
**Light**, in general, from: acon. agar. ars. bufo. cact. cocc. euphr. sep. sil. tabac. ziz.
— from artificial: bufo. croc. glon. mang. sang.
— from daylight: nat-m. sil.
— from working under gas: glon. nat-s.
**Looking fixedly** at anything, from: cadm. cina. gent. glon. helon. lith. mur-ac. par. puls. spong. sulph. tarent.
— — relieves: agn. sabad.
— **downward**, from: nat-m. phyt.
— — out of window causes vertigo, anxiety, headache, and sweat: oxal-ac.
— **upward**, from: acon. æth. arn. arum-t. caps. coca. colch. glon. gran. puls. stram. sulph. thu.
— **sideways**, from: acon.
— — relieves: olnd.
**Lying**, when: agar. alum. ambr. am-c. anac. ars. aur. bar. *bell.* bov. cadm. calc. camph. carb-v. cham. cimic. clem. coloc. cupr. dios. dulc. euph. euphr.

eupi. gels. glon. hepar. ign. k-ca. lac-can. led. lith. lyc. mag-c. mag-m. mang. men. merc. mezer. mur-ac. nit-ac. nx-v. petr. ph-ac. phys. plat. puls. ran-b. rhod. rhus. sang. sep. spig. spong. stann. staph. stront. sulph. therid. znc.
— — must stand or walk: chin.
— **relieves**: alum. ambr. am-m. anac. arn. asar. bell. benz-ac. bry. bufo. calc. calc-p. camph. canth. chel. chin. chin-s. cocc-c. colch. con. dig. dulc. ferr-i. fluor-ac. ham. hell. hipp. ign. k-bi. k-ca. lach. lyc. mag-c. merc. mur-ac. nat-c. nat-m. nit-ac. *nx-v.* olnd. petr. phos. ph-ac. sabad. spig. sulph. tabac. znc. ziz.
— **forehead**: alum. arg. bov. bry. calc. camph. cinnb. fluor-ac. lachn. mag-s. merc. ran-b. tong.
— — relieves: anac. bell. calc. con. glon. ham. k-bi. meli. pip-m. rhus. sep. spig. tabac.
— **occiput**: camph. canth. chel. euph. lachn. mag-s. nx-v. pip-m. puls. spig. staph.
— — relieved: alum. graph. iod. nit-ac. spig. tabac.
— — — on the part: ph-ac. sep.
— **side**: carb-v. petr. rhod. sep. spong.
— — relieves: dig. ment-pi.
— **temple**: camph. calen. clem. graph. lith. mag-m.
— — relieves: asar. benz-ac. chel. chin-s. colch. ferr. mag-c. nx-v.
— **vertex**: chel. hipp.
— — relieves: calc-p. spig.
**Lying** on abdomen, amel. occipital pain: grat.
— **on back**, when: ailan. bry. cact. cinnb. coloc. ign. nx-v. petr. plect. sep. spig.
— — — of head, aggr.: cact.
— — — relieves: bry. ign. nx-v. par. petr. puls. verat.
— **on side**, when: cact. calad. cocc. ign. graph. ign. nx-v.
— — — relieves: ign. men. sep.
— **on right side**, when: brom. staph.
— — — — relieves: brom. cinnb. nx-v.
— **on left side**, when: cinnb. nx-v.
— **on painful side**, when: ars. calad. calc. carb-v. graph. mag-c. mag-m. *nx-v.* ph-ac. puls. *spong.* stann. staph.
— — — — relieves: arn. hipp. ign. nx-v. puls. sep.
— — side pains on which one lies: calad. graph. mag-c. ph-ac.
**Masticating**. See Chewing.
**Meals**. See Eating, Breakfast, etc.
**Measles**, after: bell. dulc. hell. hyos. puls. rhus. sulph.
**Menses**, before: *acon.* alum. asar. *bell.* *borax.* *bov.* *brom.* bry. calc. calc-p. carb-an. carb-v. *cimic.* *cinnb.* creos. cupr. ferr. *gels.* glon. hepar. hyper. iod. *lach.* merc. nat-c. *nat-m.* nit-ac. nx-m. nx-v. petr. *plat.* *puls.* sil. *sulph.* *verat.* vibur. xanth. znc.
— — at commencement of: hyos.
— — **relieved**, when the flow begins: all-s. alum. lach. verat.
**Menses, during**: acon. agar. aloe. alum. am-c. am-m. ant-c. apis. arg-n. ars. asar. bell. berb. borax. *bov.* brom. *bry.* bufo. calc. canth. carb-an. *carb-v.* cast. caus. cham. chin. cic. cocc. con. *creos.* cub. cupr. cur. cyc. eupi. ferr. gels. gent. *glon.* graph. hyos. hyper. *ign.* k-bi. k-ca. lach. *laur.* *lyc.* *mag-c.* mag-m. mag-s. *nat-c.* *nat-m.* nit-ac. nitr. *nx-m.* *nx-v.* *phos.* *plat.* *puls.* rhod. sang. sep. sil. stann. *sulph.* *verat.* xanth. znc.
— — **relieved**: bell. verat.
— — **forehead**: æsc-h. am-c. apis. bell. brom. bry. cast. gels. helon. iod. k-bi. lyc. mag-c. merc. nat-c. nat-m. nx-v. phos. plat. ratan. sang. sep. sulph.
— — **occiput**: carb-an. mag-c. mag-m. nitr. nx-v.
— — **side**: am-m. berb. mag-c. mag-m. nat-c. sang.
— — **temple**: am-m. cast. lyc. nat-c.
— — **vertex**: calc. carb-an. cast. lach. laur. lyc. mag-c. nx-v. ol-an. phos. ratan. sulph.
**Menses, after**: agar. calc-p. carb-ac. carb-an. *ferr.* glon. k-bi. lach. lyc. nat-m. ol-an. plat. puls.
— — as if top would fly off: ustil.
— — morning, on awaking, after sudden cessation of: lith.
**Mental emotion**, after. See Excitement.
— **labor**, from: acon. agn. ambr. am-c. *anac.* aran-d. arg-n. arn. asar. aur. bell. cadm. calc. cham. chin. cimic. cina. cinnb. cocc. coff. colch. con. daph. dig. fago. graph. hell. hipp. ign. lach. lact. lyc. mag-c. nat-c. nat-m. nat-s. nit-ac. nx-m. nx-v. olnd.

op. oxal-ac. par. petr. phos. ph-ac. plat. psor. ptel. puls. sabad. selen. sep. sil. staph. stram. sulph. znc.
— — see also under Reading, etc.
— — **relieves** : ham. helon. phys. pip-m.
— — **forehead** : arn. asar. calc-ac. coff. cop. dig. fago. hydrs. hydrph. kalm. lact. manc. meli. mezer. nat-m. ol-an. ph-ac. pip-m. psor. puls. rhus-r. sil. tereb.
— — **occiput** : anac. aster. colch. ign. lobel. nit-ac. rhus-r.
— — — relieves : cact. calc-ac.
— — **sides** : hyper. ign.
— — **temples** : dig. gent. hell. nat-c. nx-v. ph-ac. pip-m. psor. sulph.
— — — amel. : calc-ac.
— — **vertex** : aster. gent. *pic-ac.* nx-v. sep.
**Mercury**, after abuse of : asaf. aur. carb-v. chin. fluor-ac. *hepar.* iod. k-iod. mezer. *nit-ac.* podo. puls. sars. staph. still. sulph.
**Metallic** substances, from abuse of: sulph.
**Milk**, after drinking : brom. phys.
**Motion**, from : acon. agn. aloe. ambr. am-c. am-m. anac. ant-t. apis. arg-n. arn. ars-i. aur. bapt. *bell.* berb. bism. bov. *bry.* bufo. calc. calc-p. camph. cann-s. canth. carb-v. caus. *chin.* chin-s. cimic. cob. cocc. colch. coloc. croc. cupr. dulc. fago. fluor-ac. gels. gent. glon. graph. grat. hepar. hydrph. ign. indm. iod. k-ca. kalm. lach. laur. led. lobel. lyc. mag-c. mag-m. mang. merc. merc-s. mezer. mosch. naja. nat-c. *nat-m.* nicc. nitr. nx-j. nx-m. *nx-v.* petr. phos. ph-ac. phys. pic-ac. plat. ratan. rheum. rumx. sabad. samb. sang. sars. sep. sil. *spig.* spong. stann. *staph.* sulph. thea. *therid.* thu. verat. zing.
— see also Motion of Arms, Eyes, Head, Exertion, Walking, etc.
— **relieves** : alum. am-m. ant-t. calc. caps. cham. cina. coloc. dros. euph. guai. hipp. hyos. k-iod. mag-c. mag-m. men. mosch. mur-ac. op. petr. phos. puls. rhod. rhus. ruta. seneg. sulph. tarax. valer. verb.
— **forehead** : acon. agn. ang. ant-t. ars. atro. aur. aur-m. *bell.* bism. bov. *bry.* calc. calc-ac. canth. cinnb. cupr. cyc. dig. dulc. fago. glon. graph. ign. iod. k-bi. lyc. mag-c. meli. men. mosch. nat-c. nx-j. ph-ac. phys. rhod. rumx. sabad. sep. sil. sol-n. spig. staph. *sulph.* tabac.
— — rapid : dros. nat-m.
— — relieves : hydr. lach. pip-m.
— **occiput** : aur. bism. *bry.* chin. colch. cupr. gels. hyper. iod. lac-ac. man. *mezer.* ph-ac. spig. spong.
— — relieves : carl. pip-m.
— **sides** : agn. arg-n. bell. calc-p. chin. dirc. glon. hipp. iris. phos. ph-ac. prun. sabad. sil. spig.
— **temple** : agn. caus. chin. cinnb. cob. cupr. dirc. glon hipp. hydrs. k-bi. *mezer.* phos. ph-ac. phys. rhod. thu. znc.
— — relieves : carl. com. lil-t. mezer.
— **vertex** : aur. bell. calc-p. canth. chin. ferr. glon. hydrph. ipec. iris. lobel. mezer. ph-ac. phyt. sep. spig. thu. verat.
**Mouth**, by opening, aggr. : fago. spig.
**Move**, on beginning to : iris. therid.
— — — relieves : valer.
— from violent : calc dros. mezer.
— — — relieved : sep.
— from quick : coral. nat-c. petr.
— on moving in bed : colch.
— pains compel one to : chin. ph-ac.
**Music**, from : acon. ambr. nx-v. phos. *ph-ac.* viol-od.
**Narcotics**, after abuse of : bell. cham. coff. dig. graph. hyos. lach. lyc. nx-v. op. puls. sep. valer.
**Nervous** : acet-ac. acon. agar. agn. ailan. anac. apis. arg. arg-n. arn. ars. asaf. asar. asc-t. atro. aur. bell. bry. cact. calad. calc. camph. cann-s. caul. caus. cedr. cham. chin. chlor. cic. cimic. cina. coca. cocc. coff. coloc. croc. croton. form. gels. glon. graph. hydrs. hyos. ign. ipec. iris. lact. nat-m. nx-v. op. petr. phos. ph-ac. plat. puls. rhus-r. rhus. sang. scut. sep. sil. spig. stict. stram. sulph. tarent. tereb. therid. thu. ustil. valer. verat. verat-v. znc.
**Noise**, from : acon. anac. ang. arn. ars. bapt. bar. *bell.* bufo. cact. calad. calc. cann-s. colch. *con.* hell. hyos. ign. iod. lac-can. merc. nat-c. nit-ac. nx-v. *ph-ac.* sil. *spig.* stann. tabac. THERID. yuc. znc.
— — of distant talking : mur-ac.
— — of hammer on anvil : manc.
— — of falling water : nit-ac.
— — of rattling of vehicles : nit-ac. therid.

— **forehead**: acon. agar. cact. cit-v. colch. con. iod. plect. spig.
— **occiput**: ph-ac. spig.
— **side**: cact. ziz.
— **vertex**: cact. iod. spig.
**Nose**, bleeding of, relieves: ant-c. bufo. dig. ferr-p. hyos. k-bi. *meli.* mill. tabac.
— **blowing**, when: ambr. aster. chel. ferr. mur-ac. nit-ac. sulph.
— **odors**, from strong: acon anac. *aur. bell.* cham. chin. *coff. colch.* graph. *ign. lyc.* nx-v. *phos.* selen. *sulph.*
— — of alcohol: sol-t-æ.
— — of coffee: lach.
— — of dirty clothes: carb-an.
— — strong and agreeable: arg-n.
**Nursing** infants, after: bell. bry. *calc.* cham. chin. dulc. phos. *puls. sep.* sil. staph.
**Opium**, from abuse of: acet-ac.
**Overheating**. See Heated.
**Parturition**, during, lying-in, etc.: arn. *bell.* bry. calc. carb-an. *cham.* coff. ferr. hyos. k-ca. nx-v. plat. *puls. rhus. sep.* sulph.
— **pregnancy**, during: *bell.* bry. calc. caps. caus. *cham.* cocc. hyos. nx-m. plat. *puls.* rhus. *sep.* sulph.
**Periodical** headache: æth. aloe. ambr. anac. arn. *ars.* asaf. bell. benz-ac. calc. cedr. cham. chin. chin-s. creos. cupr. ferr. k-bi. laur. lobel. mur-ac. nat-c. nat-m. nat-s. nicc. *nx-v.* phos. plat puls. rhus. sang. selen. sep. sil. spig. sulph.
— aggr. periodically: cupr.
— every day: nx-m. sabad. sulph.
— — — from 9 to 1: mur-ac.
— — morning: hepar.
— — from 4 P. M. to 3 A. M.: bell.
— — other day: ambr. cimic. merc-c. phos. psor.
— — — morning on awaking: eup-per.
— — fortnight: nicc. sulph.
— — six weeks: mag-m.
— — seven days: phyt. sang. sil. sulph.
— — eight days: iris.
— periodic headache with vertigo and nausea, morning on awaking, also in evening: often relieved by pressure, in open air or by eating: k-bi.
— — in afternoon, increasing until midnight; every third attack alternately more or less violent: lobel.
**Perspiration**. See Sweat.
**Pressure, external**, aggr.: agar. am-c. ant-c. arg. bell. bism. bry. calc. camph. castor. chin. *cina.* cinnb. cupr. glon. *k-ca.* lach. lact. lyc. mag-c. mag-m. marum. merc-i-fl. mezer. mur-ac. nat-c. ph-ac. *prun.* sabin. sars. sulph. valer. verb.
— cannot bear pressure though it does not aggravate: seneg.
— **relieves**: alum. am-c. anac. ant-c. apis. arg-n. bell. bry. calc. camph. carb-an. chel. *chin.* cimic. cinnb. con. *glon.* guai. hepar. indm. ipec. k-bi. lach. laur. mag-c. mag-m. men. merc. merc-i-fl. mezer. mur-ac. nat-c. *nat-m.* nat-p. nicc. nitr. nx-v. olnd. par. phos. puls. ran-sc. sabad. sabin. sep. sil. spig. *stann.* staph. sulph. sul-ac. tarent. thu. verat.
— of a cold hand, relieves: calc.
— hard, relieves: anac. bell. carb-an. *chin.* mag-m. men. nx-m.
— **forehead**: calc. camph. dios. mag-m. marum. mur-ac. ph-ac.
— — relieves: ailan. am-m. anac. aral. bell. calc-ac. carb-ac. castor, chel. chin. cimic. clem. colch. croc. ferr. gels. glon. ham. hell. hydrs. ipec. k-iod. lil-t. mang. men. merc. mur-ac. nat-c. nat-m. olnd. op. phys. sabad. spig. stann. sulph. sul-ac.
— **occiput**: calc. camph. dios. ph-ac. sulph.
— — relieves: calc-ac. colch. grat. hydrs. hyos. mag-c. mang. man. nx-v. sabin. sep. spig. tarent.
— **side**: agar.
— — relieves: mezer. sulph.
— **temple**: aspar. bism. castor. cina. cop. daph. lil-t. mur-ac. nat-ars. nat-m. nitr. prun. verb.
— — relieves: æth. alum. ant-c. aral. cact. calad. calc-ac. chin. cocc-c. cop. dios. dirc. glon. guai. hydrs. iod. k-iod. lil-t. mag-c. mag-m. men. nat-c. par. phos. plan. podo. stann. thu. verat.
— — — by pressure on opposite side: jac.
— **vertex**: bell. castor. cina. k-ca. lach. nat-c. nitr.
— — relieves: cact. dirc. eup-per. ferr. ph-ac. phys. stann.
**Quiet**. See Rest.
**Reading**, aggr. from: agn. apis. arg. arn. aur. borax. bov. bry. *calc.* calc-ac. carb-v. caus. chin-s. cina. cinnb.

clem. coca. cocc. coff. croton. ferr-i. glon. helon. *hydrph.* ign. lach. lyc. merc. mezer. nat-m. nat-s. nx-v. olnd. op. par. ruta. sabad. sil. sulph.
— see also under Mental labor, Writing, etc.
— relieves: ham. ign.
— **forehead**: arn. borax. bry. *calc-ac.* caus. chin-s. cocc. coff. ferr-i. hydrph. lob-s. op. phys. pip-m. tarax.
— **temple**: calc-ac. clem. coca. mezer. nat-m. phys. pip-m. sulph.
— **vertex**: carb-v. helon. lyc. nat-m.
**Rain**, amel.: cham.
**Rest**, aggravates: arg. asar. benz-ac. caps. cham. cic. coff. euph. ferr. lach. lyc. mang. men. nat-c. nitr. puls. rhod. rhus. ruta. samb. stann. staph. valer.
— **relieves**: ant-c. arg. arg-n. arn. *bry.* chel. cic. cocc. colch. croton. cupr. eupi. gent. glon. *hell.* hepar. hipp. iod. k-bi. k-ca. lac-can. laur. lyc. nat-m. nitr. *nx-v.* olnd. phos. rhod. sang. sars. sep. spig. squil. staph.
— compare with Lying, Sitting, etc.
**Retching**. See Eructation.
**Rheumatic**: acon. am-m. asc-t. bell. berb. bry. calc-p. caul. caus. cham. chin. cimic. coloc. dulc. guai. ign. k-bi. kalm. lach. led. lyc. mag-m. merc. nit-ac. nx-v. phos. phyt. podo. puls. rhus-r. rhus. sep. spig. stict. stram. sulph.
**Riding**. See Driving.
— in cars, aggr.: coloc. k-ca.
**Rising**. See under Morning, Noon, Night, etc., on Rising; also under Head, raising.
**Rising, after**: arn. glon. laur. nat-m. oxal-ac. phos. stram. tarent.
— relieves: asaf. hepar. ign. k-iod. merc. nat-c. nat-s. nit-ac. rhus-r.
— **forehead**: cob. dulc. glon. iber. kalm. mur-ac. phys. sang. verat.
— — relieves: chin-s. cinnb. spong.
— **occiput**: gels.
— — relieves: chin-s. eup-per. grat. jug-c. puls.
— **temple**: fago. lycps. verat.
— — relieves: calc-ac. rhus. stann.
— **side**: chin-s. graph.
— — relieves: carb-v. dig. indg. ol-an. rhod. tabac. tong.
**Rising from lying**, aggr.: æsc-h. am-m. anac. ang. apis. ars. asar. bapt. bell. bov. bry. calc. camph. caps. carb-an. cham. chel. cinnb. clem. coca. coloc. con. coral. *dulc.* glon. hepar. ipec. mur-ac. nat-c. nitr. nx-v. olnd. phos. ph-ac. puls. rhod. ruta. sep. sil. squil. sulph. staph. ustil.
— — — **relieves**: aloe. ambr. am-c. ars. aur. bell. calad. carb-an. carb-v. cham. chin. cic. cup-ac. ferr. gels. hepar. ign. k-ca. laur. lith. mag-c. nat-m. nit-ac. nitr. nx-v. phos. ph-ac. phys. plb. puls. ran-b. rhod. rhus. sabin. spig. verat.
— **from sitting**, aggr.: apis. bell. chin. cob. ferr. grat. lam. laur. lyc. mang. mur-ac. oxal-ac. puls. sil. spong. trio. verat.
— — — relieves: arg. phys. spig. spong.
— **from stooping**, aggr.: acon. asar. coral. daph. hepar. k-ca. lam. lyc. mag-m. mag-s. mang. mur-ac. nx-v. sul-ac. tong. viol-tr.
— — — relieves: calc-ac. con. ign. indg.
— — — forehead: asar. mag-s.
— — — side: k-ca. mang. sul-ac.
— **upright**, erect, aggr.: acon. ang. arn. ars. asar. bell. bov. bry. caps. caus. cham. cic. dros. hell. hepar. ign. k-ca. laur. lyc. mag-m. mang. mur-ac. spong. sul-ac. tarax. verat. viol-tr.
— — relieves: ant-t. cic. mag-c. rhus. sabin.
— **standing position**, rising to, relieves: alum. ang. aur. bar. bry. calc. canth. carb-v. chin. con. dig. k-ca. laur. mag-c. marum. nat-c. olnd. puls. rhus. spig. stann.
**Rocks** head from side to side to relieve pain: k-iod.
**Room**, aggr.: æth. all-c. am-m. arn. ars. asaf. bov. bry. calc-ac. caus. cham. chel. *cimic.* cob. coca. colch. croc. euphr. ham. hepar. hipp. hyos. iod. jatr. laur. mag-c. mag-m. *mang.* men. mezer. mosch. nat-c. nicc. nx-v. phos. ph-ac. phys. plat. *puls.* ran-sc. rhod. sabad. seneg. sep. sulph. tilia. ton. verat. verb. znc.
— pains coming on in room are relieved out-doors, and *vice versa:* mang. ran-b.
— **relieved** in: bell. bry. bov. cham. chel. chin. cocc. *coff.* eup-per. ferr. hepar. mag-c. mang. merc. nx-m. *nx-v.* rhus. spig. staph. sulph. sul-ac. valer.

— **forehead**: acon. bry. cact. caus. coca. colch. con. lach. nx-j. plat. ran-b. rhod. rhus. sep.
— — relieved in: bell. mang.
— **occiput**: bry. cimic. mag-m. mezer.
— — relieved: bov.
— **temple**: jatr. laur. phos. ran-b. rhod. sabad.
— — relieved: coff. hyos. ol-an. zing.
— **side**: am-m. bov. euphr. fluor-ac. phos. sabad. ton.
— — relieved: mag-s.
— **on entering** a, aggr.: mezer. nat-m. *ran-b.* ran-sc. rhus. sabad. spong.
— — — from cold: colch. con. puls.
— **warm** room, aggr.: all-c. aloe. apis. *arn.* bar. bov. cann-i. caus. cocc-c. croc. ferr-i. hydrs. lact. laur. lil-t. mezer. merc-i-fl. nat-c. nitr. phos. ph-ac. plat. *puls.* selen. seneg. sin-n. sol-n. spong. sulph. tanac. znc.
— — compare with Air, warm.
— — relieves: am-c. lac-can. nx-v. sil.
— — **forehead**: acon. bov. carb-an. caus. lac-ac. lil-t. mezer. plat. selen. seneg. sin-n. verb.
— — — relieved: lac-can. sil.
— — **occiput**: bov. *seneg.*
**Rubbing**, aggr.: alum. calc-p. caus. dios.
— **forehead**, relieves: ars. ham. ol-an. op. phos. phys.
— **occiput**, relieves: canth. laur. ol-an.
— **temple**, relieves: canth. ol-an. phos. plat.
— **vertex**, relieves: carb-ac.
**Running**, from: bry. nat-m. nx-v. tarent.
**Scarlatina**, after: am-c. bell. bry. cham. dulc. hell. hepar. lach. merc. rhus.
**Scratching**, amel.: mang.
**Sexual excesses**, after: agar. arn. *calc.* carb-v. chin. con. k-ca. lach. merc. nat-c. *nx-v.* phos. ph-ac. pip-m. puls. *sep.* sil. spig. *staph. sulph.* thu.
— — after onanism: *calc.* carb-v. *chin. con.* lyc. merc. nat-m. nx-v. phos. puls. *sep.* spig. *staph.* sulph.
— — after excessive pollutions: alum. *calc.* con. ham. *k-ca.* lyc. *nx-v.* sep. staph. viol-od.
— — occipital pain: *chin.*
**Sewing**: lac-can.
**Shade**, compare Darkness and Sun, aggr. in.
**Shame**, from: op.
**Siesta**. See under Sleep.
**Singing**, from: alum.
**Sitting**, aggr. when: agar. alum. am-m. ang. aral. arn. ars asaf. asar. bism. borax. bry. bufo. calc. canth. carb-an. caus. cham. *chin.* cic. coff. con. cyc. dros. euph. gent. grat. guai. indg. ferr. lach. led. lyc. mag-c. men. merc. merc-i-r. mezer. mosch. mur-ac. nat-c. *phos.* plat. puls. ran-b. ratan. rhod. rhus. ruta. sabad. seneg. sil. spong. squil. *staph.* sulph. sul-ac. tarax. verat. zing.
— — up or erect. See under Rising.
— **relieves**: ars. asar. calc. calad. coff. con. gels. glon. hipp. ign. k-ca. lam. mag-m. merc. nx-v. sulph. verat.
— **forehead**: æth. agar. alum. am-m. bism. calc-ac. castor. caus. *chin.* con. glon. ham. lach. merc. mezer. phos. ruta. seneg. spig. spong. staph. tarax. tereb. verat.
— — relieves: acon. ars. bell.
— **occiput**: agar. castor. chin. indg. men. ran-b. spig. squil. znc.
— — relieves: asar. ign. mag-c. mag-m.
— **side**: am-m. canth. chin-s. fago. indg. mag-c. nicc. phos. ratan. rhod. sulph.
— — relieves: ars. calad. con.
— **temple**: am-m. arg. chin. lil-t. mang. mezer. nicc. phos. staph. sul-ac. tarax. verat.
— — relieves: ars. asar. calc-ac. coff. coloc. lith. mang.
— **vertex**: castor. lyc. peti. phos. verat. viol-tr.
— — relieves: con. gels.
**Sleep, before** going to: agar. nx-m.
— **during**: agn. ars. camph. cham. colch. dig. ferr. graph. led. mag-c. petr.
— — second sleep, morning, aggr.: ham.
— — on falling asleep, amel.: anac. ang. nit-ac.
— — relieved during: acon. bad. glon. hell. hyos. ign. pallad. sep. sil. thu.
— **on awaking**, aggr.: agar. ailan. alum. ambr. anac. *ant-t.* arg-n. ars. aur. bar. bell. bov. *bry.* cadm. calad. calc. carb-an. carb-v. caus. cham. chel. chin. chin-s. cic. cimic. cina. cinnb. clem. cocc. coff. con. croc. dig. dros. erig. euphr. gels. graph. ham. hell. hepar. ign. ipec. k-bi. k-ca. lach.

lact. lyc. mag-c. mag-m. mill. morph. nat-c. *nat m.* nat-s. nitr. nit-ac. nx-m. *nx-v.* op. oxal-ac. par. petr. peti. ptel. phos. ph-ac. plect. plb. psor. puls. raph. rheum. rhus. rhus-r. rumx. ruta. sabad. selen. sep. sil. squil. staph. stram. sulph. sul-ac. tarent. thu.
— — after restless sleep : crot-c. stram.
— — see also Morning, Night, on waking.
— — amel. on awaking : bell. camph. chel. glon. graph. ham. laur. phos. sang. thu.
— — — by a good sleep : sep.
— **siesta**, after a : bov. calad. calc-s. carb-v. coff. ign. merc-i-r. nx-m. rhus. sep. sulph.
— — relieved : nitr.
— **loss** of, from late hours, aggr. : ant-c. carb-v. coff. colch. *laur. nx-v.* rhus. sulph.
— — from night watching : ambr. bry. carb-v. *cocc.* colch. *nx-v. puls.*
**Smell**. See Odors under Nose.
**Smoking**. See under Tobacco.
**Sneezing**, when : am-m. arn. bar. bry. cina. grat. k-ca. *nat-m.* sabad. sulph.
— — relieves : calc. lil-t. mur-ac.
**Soup**, from warm : k-bi.
**Speaking**. See Talking.
**Spinning**, from : carb-an.
**Spirituous liquors**, from abuse of : acet-ac. agar. ant-c. ars. asaf. bell. bry. calc. carb-v. chel. chin. cimic. coff. hell. hydr. ign. ipec. *lach.* lyc. merc. nat-m. nit-ac. nx-m. *nx-v.* op. phos. puls. *ran-b.* rhod. rhus. ruta. sabad. selen. sil. spig. stann. *sulph.* verat.
— — see also Alcohol, Beer, Wine, etc.
— — relieve : arg-n. bufo. castor. hell. naja. phos. sep.
**Standing**, aggr. when : agar. alum. arg. arn. ars. calc-ac. canth. chin. dig. guai. mag-c. mang. puls. ran-b. rheum. rhus. staph. sulph. tarax. verat. znc.
— relieves : calc. camph. ran-b.
— **forehead** : agar. alum. ars. calc-ac. canth. chin. ham. k-ca. merc. phel. ran-b. rheum. sang. spig. staph. tabac. tarax.
— — relieves : calc-ac. marum.
— **occiput** : castor. k-ca. mag-c. tabac.
— — in one position, when : cham.
— — **relieves** : nx-v. plb.
— — — when standing still : tarax.
— **side** : calc. canth. dig. k-ca. mag-c. mang. plb. znc.
— **temple** : ars. castor. chin. coloc. glon. guai. staph. verat.
— — relieves : tarax. zing.
— **vertex** : ran-b. sul-ac. verat.
**Stepping**. See Walking.
— **heavily**, aggr. : alum. ambr. bell. bry. calc. calc-p. chin. cocc. dros. hell. k-ca. lach. led. lyc. men. nat-m. nit-ac. nx-v. phos. ph-ac. rhus. sep. sil. spig.
— **false**, from a : anac. bry. cob. hepar. led. puls. sol-n. spig. thu. vibur.
— **at each** step : alum. ambr. bell. bry. calc-p. cocc-c. coloc. con. dros. glon. hydrs. lach. lyc. mezer. nat-m. nit-ac. nuph. ph-ac. sep. spig. spong. sulph.
— compare with Jar, etc.
**Stomach disordered**. See Gastric.
**Stool**, from urging at : bell. calc-p. coloc. con. glon. ham. hell. indm. ign. iod. lyc. phos. ratan. spig. sulph. thu. vibur.
— from insufficient : *aloe.* con.
— **after** : aloe. ambr. apoc. bufo. chel. podo. sabad. sep. spig.
— — relieved : æth. agar. aloe. apis. corn. lachn. ptel. oxal-ac. verat-v.
**Stooping**, from : acet-ac. acon. æsc-h. aloe. alum. am-m. ang. ant-t. apis. arg. arn. asar. bapt. bar. *bell.* berb. borax. bov. *bry.* calc. calc-caus. calc-p. camph. canth. caps. carb-an. carb-v. caus. cham. chel. chin. chin-s. cic. cob. cocc. coff. colch. coloc. com. corn. creos. cupr. cyc. dig. dros. dulc. ferr. ferr-i. form. gels. glon. ham. hell. helon. hepar. hydrs. hydr-ac. hydrph. hyos. ign. k-bi. k-ca. lach. laur. lyc. mag-m. manc. mang. marum. men. merc. merc-i-r. mill. mur-ac. nat-c. nat-m. nicc. nit-ac. nitr. nx-m. *nx-v.* par. petr. phos. phys. phyt. pic-ac. plat. plect. puls. rheum. *rhus.* rhus-r. samb. *sang.* seneg. senn. *sep. sil.* spig. spong. stann. staph. sulph. sul-ac. thu. valer. verat. vibur. zing.
— **after**. See Rising from.
— heavy pressure on brain forcing to stoop : cann-i.
— **relieves** : ang. caus. *cina.* con. dig. elaps. fago. *hyos.* ign. indg. laur.

mang. mezer. nx-v. phos. tarax. verat. verb. viol-tr.
— **forehead**: acon. am-c. am-m. ang. arg-n. arn. asar. atrop. aur-m. bar. bell. berb. borax. bov. brom. *bry.* calc-ac. camph. canth. carb-an. carb-v. card-b. caus. chel. cob. coff. coloc. creos. cupr. cyc. dros. dulc. fago. fluor-ac. gels. gran. guai. hæmat. hepar. hyos. hydrph. ign. indm. ipec. junc. k-bi. k-iod. lact. laur. lyc. mag-m. manc. mang. marum. merc. merc-c. murx. mur-ac. myric. nitr. nx-v. ped. pic-ac. plat. ptel. puls. ratan. *rhus-v.* sil. sol-n. spig. stann. staph. sulph. tarent. valer. verat.
— — relieves: bar-ac. bell. caus. verb.
— **occiput**: acon. aloe. camph. carb-v. chin. colch. gels. hell. helon. k-ca. mang. ph-ac. prun. rhus-r. spig. sulph.
— — relieves: ign. ol-an. verat.
— **side**: alum. ang. calc-ac. caps. chin-s. coral. dig. euphr. glon. hipp. indg. laur. ment-pi. phos. ton.
— — relieves: dig. iris.
— **temple**: am-m. bov. brom. calc-ac. chin. coff. coloc. dios. fago. fluor-ac. glon. guai. mur-ac. nat-ars. phos. sol-n. thu. verat.
— — relieves: ang. mang. verat.
— **vertex**: acon. almn. am-m. berb. calc. calc-p. coloc. glon. helon. men. nx-m.
— — relieves: laur verat.
**Stove**, from heat of: *arn.* *bar.* cimic. puls.
**Studying**. See Mental labor.
**Sun**, from exposure to: *acon.* aloe. ant-c. bar. *bell.* brom. bruc. cadm. calc. *camph.* cann-i. castor. chin-s. euphr. gen. glon. hipp. hyos. ign. *lach.* manc. *nat-c.* nat-m. nx-v. *selen.* therid. valer.
— relieved in: graph. stram. stront.
— — in shade: brom.
**Supper**, amel. after: am-c. lachn.
— see Eating.
**Swallowing**, when: mag-c.
— see also Chewing.
**Sweat** preceded by headache: ferr.
— **suppressed**, from having: *ars.* *bell.* bry. *calc.* *cham.* *chin.* lyc. merc. *nx-v.* phos. *puls.* rhus. sep. *sulph.*
— **relieves**: bov. clem. graph. mag-m. *nat-m.* psor. *sulph.* tarent. thu.
— **during** headache: arn. ars. eup-per. nat-m. rhus. thu.
— **after**: calc. *chin.* merc. puls. *sep.* staph. sulph.
**Talking**, when, aggr.: *acon.* agar. aran-d. aur. bry. cact. calc. canth. chin. cic. cocc. coff. con. dros. dulc. euphr. fluor-ac. glon. hyos. *ign.* iod. lac-can. led. mag-m. man. merc. *mezer.* nat-m. nx-j. nx-v. par. ph-ac. puls. rhus. sang. sars. *sil.* spig. spong. sulph. znc.
— relieves: eup-per. ham.
— distant: mur-ac.
— of others: aran-d. bar. ign. merc.
**Tea**, from abuse of: chin. lach. selen. thu. verat.
— — relieves: ferr-p.
— — — by strong: carb-ac. glon.
**Teeth**, on compressing the: indm.
— — see also Chewing.
**Temperature**, from a change of: carb-v. *ran-b.* verb.
— see also under Weather.
**Thinking** of pain, aggr.: cham. chin. ferr-p. hell. helon. ign. pip-m. sabad. sin-n. staph.
— — — relieves: agar. camph. *cic.* prun.
— see also Mental labor.
**Thunder-storms**, air just before, aggr. from: bry. lach. *nat-c.* phos. *rhod.* *sep.* *sil.*
— — during, aggr.: nat-p.
**Tobacco**, smoking, from: acet-ac. *acon.* *ant-c.* calc. caus. clem. cocc. cocc-c. ferr. gels. glon. *ign.* mag-c. nat-ars. nat-m. nx-v. op. par. puls. spig. thu.
— — relieves: am-c. aran-d. calc-p. carb-ac. naja.
— — **forehead**: calad. caus. ferr-i.
**Touch**, aggr. by: *acon.* agar. agn. all-c. alum. arg. bar. *bell.* borax. bov. bry. calc. camph. casc. cast. carb-an. chel. *chin.* cinnb. con. cupr. daph. grat. hydrph. ipec. k-bi. k-ca. lact. laur. led. lyc. mag-s. merc. mur-ac. nat-m. nit-ac. nitr. nx-m. par. phos. ph-ac. rhod. sars. sep. spig. sabin. staph. tarent.
— — on vertex, from: sabin.
— **relieves**: ars. asaf. bell. bry. calc. coloc. con. cyc. mang. men. mur-ac. nitr. phos. sars. thu. viol-tr.
— compare with Pressure.
— **brain**: all-c. arg. bry. chin. cinnb. grat. k-bi. lact. laur. mag-s. merc. merc-i-fl. mezer. mur-ac. nat-m. par. sabin. sars. staph. sulph. viol-tr.

— — relieves: sars.
— **forehead**: chin. cupr. ipec. lepi. lyc. mur-ac. nat-m.
— — relieves: bell. calc-ac. chin. cyc. mur-ac. viol-tr.
— **occiput**: bar. cupr. nit-ac. nitr.
— — relieves: mang.
— **side**: agar. agn. borax. cupr. dirc. laur. nit-ac.
— — relieves: bry. thu.
— **temple**: aur. berb. castor. chel. con. cupr. daph. led. lepi. nx-m. peti. staph.
— — relieves: ars. calc-ac. chin. cyc.
— **vertex**: bov. chel. cinnb. k-bi. peti. phos.
— — by laying hand on it, amel.: nitr.
**Treading**. See Stepping.
**Turning** body, when: cham. glon. graph. lyc. merc-i-fl. nat-m. plan. sil.
— — in bed, when: meph.
— — see also Head, turning.
**Twilight**, aggr. in the: ang. caj. puls.
— amel. in: coca.
**Uncovering** body, from: benz-ac.
— — relieves: coral.
— see also under Covering.
**Urination**, during: coloc.
— profuse relieves: gels. sil.
**Veal**, from eating: nitr.
**Vertigo**, after: *calc.*
— See Concomitants.
**Vexation**, after: acon. *bry.* castor. *cham.* cocc. ign. lyc. mag-c. mezer. nat-m. nx-v. *petr.* phos. ran-b. rhus. staph. verat.
— compare with Angry.
**Vinegar**, aggr. by swallow of: marum.
— applying, amel.: meli. op.
**Voice**, male, affect brain: bar.
**Vomiting**, aggr. when: ars. asar. bar-m. con. eugen. glon. k-bi. lach. lyc. mezer. nx-v. phyt. secale. sep. verat.
— — **after**: cham. cocc. ferr. nat-c. nx-v.
— **relieves**: asar. calc. glon. hipp. manc. op. sang. raph. stann. sul-ac.
**Waking**, on. See under Sleep and compare Waking under Morning, Night, etc.
**Walking**, when: acon. aloe. alum. anac. ang. ant-t. arn. ars. asar. aster. bar. bell. bry. cadm. calc. caps. carb-an. caus. chin. cic. clem. cob. cocc. coloc. con. corn. dig. dros. ferr. glon. gran. guai. hell. hipp. hyos. hura. ign. iod. k-ca. *lach.* laur. led. lyc. mag-c. mang. men. merc. merc-i-fl. mur-ac. nat-c. nit-ac. nitr. nx-v. olnd. ost. par. petr. phos. ph-ac. phyt. plat. ptel. puls. ran-b. rheum. rhod. rhus. sabad. sars. sep. sil. spig. spong. staph. stront. sulph. tabac. tarax. tarent. thea. therid. ustil. verat. verb. viol-tr. znc.
— compare with Motion, Stepping, etc.
— **relieves**: am-c. ant-c. aran-d. asar. borax. calc. canth. caps. cham. chin. coca. coloc. dros. fago. glon. ham. hyos. lyc. mag-c. mang. mur-ac. nat-c. nat-m. phos. puls. ran-b. rhus. seneg. sep. sin-n. spig. staph. sulph. tarax. thu.
— **forehead**: acon. anac. arn. ars. bry. calc. calc-ac. chin. clem. coca. cocc. coloc. euphr. gran. indm. k-bi. lept. mag-c. naja. nitr. ped. peti. phys. puls. ratan. rhus-v. sars. spong. sulph. ustil. viol-tr.
— — relieves: calc-ac. chin. coca. dros. puls. ran-b. rhod. sang. staph.
— **occiput**: asar. bell. bry. calc. chin. coca. con. graph. k-ca. mur-ac. ped. phys. spig. sulph. tarax.
— **side**: arg. arg-n. ars. bell. calc. clem. con. k-ca. nat-m. plb. spig.
— **temple**: ars. asar. bufo. castor. chin. coloc. con. dios. gen. glon. hell. k-bi. lil-t. mang. phos. ran-b. sulph.
— — relieves: chin. guai. staph. tarax.
— **vertex**: carb-an. calc. cedr. con. glon. hura. peti. phyt. spong. sulph.
— — relieves: peti. sang.
**Walking in open air**, when: acon. alum. am-c. ant-c. arn. atrop. bell. borax. bov. bry. *calc.* caus. chin. chin-s. cina. coff. con. dulc. euphr. ferr. grat. hell. hepar. ign. k-ca. lam. laur. lyc. mang. merc. mur-ac. nat-m. nicc. nx-m. *nx-v.* par. petr. plat. puls. ran-b. *rhus.* sabad. sars. selen. spig. *spong.* staph. stront. sulph. sul-ac. tarax. znc.
— — — **after**: am-c. bell. bov. calc. caus. chel. chin. coca. coff. ferr. hepar. k-bi. mezer. mur-ac. nicc. nx-v. pallad. petr. puls. ran-b. ran-sc. rhus. sabad. spig. spong. znc.
— — — **relieves**: æth. ambr. am-c. ang. ant-c. aral. ars. asar. bov. canth. carb-an. caus. chin-s. cina. coff. coloc. coral. croc. diad. eup-pur. fago. gen.

glon. hepar. hyos. lach. laur. lith. lyc. mag-c. mang. mosch. nat-m. olnd. phel. phos. *puls.* ran-b. rhus. sars. seneg. sep. sulph. thu. viol-tr.
— — — **forehead**: acon. ant-c. asim. calc-ac. caus. chin. cina. coca. hell. hydrph. k-cy. merc. nat-m. plat. sars. spong. tarax.
— — — — relieves: borax. camph. coral. crotal. ham. hydrs. hyos. lyc. phys. scut. sep. thu.
— — — **occiput**: bov. caus. cina. ferr-p. mang. spig. staph. znc.
— — — — relieves: cimic. mang. seneg. sulph. tabac.
— — — **side**: chin-s. grat. ign. mag-s.
— — — — relieves: mang. merc-i-r.
— — — **temple**: arn. bry. coff. hyos. mang. nat-m. spig. tarax. zing.
— — — — relieves: psor. rhod.
— — — **vertex**, relieves: acon. aster. thu.
**Walking rapidly**, when: bell. bry. calc. chel. ferr-i. mang. tabac.
— — in the wind, from: chin. mur-ac. nx-v.
— **slowly**, when: hipp.
— — relieves: eup-pur. *puls.*
**Warm.** See Air, Room, etc.
**Warmth.** See Heat, Fever, etc.
**Washing**, from: *am-c. ant-c.* bell. bry. *calc.* canth. carb-v. cham. glon. lyc. merc. nit-ac. *nx-m.* phos. puls. *rhus. sep.* spig. stront. *sulph.*
— **cold water**, amel.: acon. aloe. ant-t. ars. asar. aur-m. bry. cham. cinnb. cyc. euph. indm. kalm. lac-ac. myric. nat-s. plan. psor. znc.
— of feet, relieves: nat-s.
— from washing hands: rhus-r.
**Weather**, from changes of: bry. calc-p. lach. *nx-m. phos.* psor. ran-b. rhod. *rhus. sil.* verb. vip-t.
— **cold**: acon. agar. am-c. *ars. aur.* bell. bry. calc. *camph. caps.* carb-v. *caus.* cocc. colch. con. *dulc. hell. hepar.* hyos. ign. *k-ca.* lyc. merc. *mosch.* nat-m. *nx-m. nx-v.* phos. rhod. *rhus. sabad.* sep. *stront.* verat.
— **damp cold**: *am-c.* ars. *bry. calc.* carb-an. *carb-v.* colch. *dulc.* glon. *lach.* lyc. merc. mosch. *nx-m. nx-v. rhod. rhus.* sil. stront. sulph. verat. zing.
— **dry cold**: acon. *asar.* bry. *caus. hepar. nx-v.* sabad. spong.
— **warm**, begins with the: glon. nat-s.
— **windy**, stormy, from: asar. *aur.* bry. cham. chin. lach. mur-ac. *nx-m.* nx-v. phos. puls. *rhod.* rhus.
**Wet**, from getting: ars. *bell.* bry. *calc. colch.* dulc. hepar. k-ca. led. lyc. *nat-m.* nx-m. puls. *rhus. sep.*
— — — when sweating: acon. calc. *colch.* dulc. *rhus.* sep.
— **head**, from wetting: bar. *bell.* led. phos. puls.
— **feet**, from wetting: gels. phos. *puls.* rhus. sep. *sil.*
**Wind**, from exposure to: acon. cham. chin. ham. mur-ac. nx-v.
— — **cold**: aur. lac-can. mur-ac. nx-v. rhus.
— — — riding in: ars-i. calc-i. glon. lyc.
— — **east**: psor.
— — **rough**: aur. mur-ac.
**Wine**, from abuse of: ant-c. arn. *ars.* cact. *calc.* carb-an. carb-v. coff. gels. glon. ign. lach. lyc. nat-c. nat-m. nx-m. *nx-v.* oxal-ac. *ran-b. rhod.* rhus. sabad. *selen. sil.* verat. *znc.*
— relieves: arg-n.
— **lead**, containing: *bell.* nx-v. plat. *sulph.*
— **sulphurous**: ars. merc. *puls.* sep.
— **sour**: *ant-c.* ars. ferr. sulph.
— see also under Alcohol, Beer, Spirituous, etc.
**Winking**, aggr.: *all-c.*
**Work**, from: anac. bufo.
— carrying a weight on shoulders: mag-s.
— relieves: merc-i-fl.
— when doing some disagreeable: chin.
**Worm** complaints, from: *calc.* chin. *cina.* graph. nx-v. plat. sabad. *sil.* spig. *sulph.*
**Wrapping** up. See Covering.
**Wrinkling** forehead, aggr.: nat-m.
**Writing**, from: aur. borax. calc. clem. dros. ferr. ferr-i. gent. glon. ign. k-ca. lyc. manc. nat-m. phos. ran-b. rhus. rhus-r. sil.
— **forehead**: borax. dros. ferr-i. k-ca. lyc. op.
— **occiput**: carb-an. cocc. gels.
— **vertex**: gels. nat-m. ran-b.
**Yawning**, when: agar. bar. chin. cyc. mag-c. mur-ac. nx-v. phyt.
— relieved after: nat-m. staph.

